# Comparative Animal Models of Human Viral Infections

**DOI:** 10.3390/pathogens11121395

**Published:** 2022-11-22

**Authors:** Hinh Ly

**Affiliations:** Department of Veterinary & Biomedical Sciences, University of Minnesota, Twin Cities, 1988 Fitch Ave., Ste. 295, St. Paul, MN 55108, USA; hly@umn.edu; Tel.: +1-612-625-3358

Comparative animal modeling has long served as a cornerstone for understanding the biological effects of infection by many DNA and RNA viruses. Over the years, conventional and unconventional animal models have been developed for this purpose. The goal of the Special Issue, “Comparative Animal Models of Human Viral Infections” is to gather the latest information on recent advances in the development of animal models in order to understand human viral infections and the diseases caused by them. The successful development of comparative animals of human viral infections has important implications in helping to determine the mechanisms of disease pathogenesis and pathologies and immune correlates of protection against virus infection, as well as in informing relevant strategies for vaccination, immunotherapeutic, and antiviral developments to benefit humans.

This Special Issue consists of five published articles (two reviews and three primary research articles), which were contributed by some of the leading researchers in the field. A comprehensive review article [1] written by Fujiwara and Nakamura at the National Research Institute for Child Health and Development and the Nihon University School of Medicine in Tokyo, Japan, summarizes the biology and pathogenesis of two important human gammaherpesviruses, Epstein–Barr virus (EBV) and Kaposi’s sarcoma-associated herpesvirus (KSHV) and of the strict human species-specific infections that they cause, which have hampered the development of suitable animal models for evaluating therapeutic and prophylactic strategies against these viruses. In this review article, the authors have done an exceptional job of describing innovative approaches to generate animal models for these human gammaherpesviruses that include experimental infection of laboratory animals [e.g., New World non-human primates (NW NHPs) and rabbits] with EBV or KSHV, infection of common marmosets, rhesus macaques, or cynomolgus macaques with the respective monkey-species specific gammaherpesviruses, of mice with the murine gammaherpesvirus 68 (MHV-68), and of humanized mouse models with the human gammaherpesviruses. Important insights about the principles of disease pathogenesis caused by EBV or KSHV (e.g., malignancy and autoimmunity) have been obtained by understanding the potentials and limitations of the different animal models as described in this review article. This work is of timely significance as a recent analysis of data from over ten million US military recruits over a period of two decades has provided new evidence to bolster the association of and to implicate a potential role for EBV infection in an increased risk of development of multiple sclerosis [2], which is a chronic inflammatory demyelinating disease of the central nervous system, in humans.

Hemorrhagic fevers can be caused by some significant human viruses, such as Ebola virus (EBOV), Marburg virus (MARV), and Lassa virus (LASV). These and other emerging viruses, such as the Severe Fever with Thrombocytopenia Syndrome Virus (SFTSV), can cause severe vascular leakage and other symptoms. In an original research article [3], Westover and colleagues at Utah State University characterized the progression of vascular leak and an increase in proinflammatory cytokines in blood and tissues of immunodeficient [i.e., interferon alpha receptor knockout (IFNAR KO)] mice infected subcutaneously with the HB29 strain of SFTSV. Using this informative mouse model, the authors showed that treatment of SFTSV-infected mice with a 28-amino-acid natural plasmin digest product of fibrin known as FX06 could reduce vascular permeability, but it did not significantly reduce lethality. It is noteworthy that FX06 has been shown to significantly reduce edema and vascular leakage into the lung while improving survival outcome when tested in mouse models of dengue shock syndrome and LPS-induced lung inflammation [4]. Because FX06 is not a direct virus-targeted antiviral, its beneficial effects in some cases of virus infection might be host-mediated, e.g., in reducing vascular permeability, following FX06 administration. For example, during the 2014 EBOV disease outbreak in West Africa, FX06 was used as an experimental form of treatment to prevent shock and multiorgan failure associated with the viral infection in some patients. In that study, the administration of FX06 was found to coincide with a substantial improvement in both vascular leak syndrome parameters and respiratory function [5]. The Westover’s study published in this Special Issue [3] provides additional supportive evidence demonstrating a potential beneficial effect of FX06 treatment in reducing vascular leak in viral-induced SFTS that would support further investigation of its use to treat other forms of viral hemorrhagic fever (VHF) infection.

LASV is another virus (a mammarenavirus or, simply put, an arenavirus) that can cause lethal VHF disease. Because there are currently no FDA-approved vaccines or therapeutics against this deadly virus, the development of animal models that can recapitulate clinical and pathological features of arenavirus-induced hemorrhagic fever (AHF) diseases in humans is necessary. Laboratory mice are refractory to AHF infections, and NHPs, while being a good animal model for AHFs, are limited by their high cost and ethical and other constraints [6]. As an alternative to NHPs and mice, Shieh and colleagues described, in this Special Issue, a small and affordable animal model for AHFs that is based on outbred Hartley guinea pigs infected intraperitoneally with Pichinde virus (PICV) [7], an arenavirus that is not known to be pathogenic in humans and that can therefore be handled safely in a conventional laboratory. The authors clearly demonstrated differential disease phenotypes and pathologies in animals infected with either the avirulent strain of PICV (known as P2 or rP2) or the virulent PICV strain (P18 or rP18). By performing a detailed histopathological and immunohistochemical analyses, the authors showed extensive pathological changes and relatively high levels of viral presence in different organs of animals infected with the virulent rP18 strain of PICV that mimic those from tissues of lethally infected human Lassa hemorrhagic fever disease. These findings support a role for outbred Hartley guinea pigs as a comparative model of human arenaviral hemorrhagic fever infections.

Another study published in this Special Issue [8] also attempted to use outbred Hartley guinea pigs as well as multimammate mice and New Zealand White (NZW) rabbits as comparative animal models for infection by Zika virus (ZIKV), which is a mosquito-borne flavivirus. As previously mentioned, NHPs are naturally susceptible to many of the same viruses (e.g., LASV and ZIKV) that infect humans, but they are relatively expensive and require specialized veterinary care and facilities. On the other hand, mice have been widely used for modeling ZIKV infection, yet there are few ZIKV-susceptible immunocompetent mouse models. It is noteworthy that LASV and other viruses, such as alphaviruses, bunyaviruses, and flaviviruses naturally infect multimammate mice *(Mastomys natalensis)*, which serve as the natural reservoir for these viral pathogens [9]. Likewise, while NZW rabbits have been shown to be susceptible to another mosquito-borne flavivirus, i.e., West Nile virus, they have not yet been examined for susceptibility to ZIKV infection. On the other hand, while outbred Hartley guinea pigs have been successfully used as a comparative animal model for congenital ZIKV infection, only those that are immunocompromised (e.g., young or pregnant animals) have been found to be most susceptible [10]. The authors of a study published in this Special Issue [8] found that the multimammate mouse and NZW rabbits are not susceptible ZIKV infection, even when infected by the natural route, i.e., via ZIKV-infected mosquito bite, or subcutaneously. They also found that none of the sexually mature (adult) male outbred Hartley guinea pigs were susceptible to ZIKV infection, which was in sharp contrast to other published findings of young and pregnant guinea pigs being susceptible to ZIKV infection [10]. These findings clearly demonstrated that there are some limitations to the use of some outbred animal species (e.g., NZW rabbits, Hartley guinea pigs, and multimammate mice) as comparative animal models of ZIKV infection. These might partly be due to the natural tendency of these animals to resist ZIKV infection or might be influenced by other natural or experimental factors, such as the pathogen dose used in the experimental viral infection.

As described in a comprehensive review article in this Special Issue by B. M. Warner [11], a research biologist working in the Special Pathogens Program at the Public Health Agency of Canada and studying those animal models described above, one of the critical aspects of comparative animal modeling is the pathogen dose used, which is typically employed at a relatively high challenge dose to ensure death in the control group in order to ascertain statistical power of the performed experiments. Additionally, during initial model characterization, multiple routes of infection are sometimes used, such as subcutaneous injection or via mosquito bite, as described in the ZIKV study [8]. However, for ease and uniformity, only a specific route of infection is often used in subsequent studies (e.g., subcutaneous injection used in the SFTSV and ZIKV studies [3,8] or intraperitoneal injection (IP) used in the PICV study [7] of this Special Issue). In many other cases and in general, IPs for rodents and intramuscular (IM) injections for NHPs are often used as the common routes of virus infection. As the author of the review article pointed out [11], both the dosage of the pathogen used and its route of administration into the animals can exert a differential impact on the host immune (antiviral) response and infection kinetics that can produce different lethality rates, mean times to death, and induction of host responses. While the author recognizes that the use of pathogen doses closer to the dose of the infection, to cause 50% of lethality (i.e., LD50), would require a greater number of animals in each experiment, which may not be ethically or economically feasible, he advocates for careful consideration of the dose of virus used in experiments that is more closely mimicking natural infection, as well as other factors in various studies. Differences in virus stock production and its long-term storage, as well as the cell types used for virus propagation, virus titer calculation methods, or methods used in determining virus-induced cytopathic effect, could all influence the in vivo infection outcomes. He has also correctly pointed out in the article that in many cases, the infectious and/or lethal human doses of a particular viral pathogen are not necessarily known or have not been well characterized, which can make a direct comparison with animal models difficult. These are some variables that make it challenging to establish a direct comparison between experimental animal and natural human viral infections. While these and other limitations of comparative animal modeling inherently exist, an awareness of how these factors might influence infection outcomes, a consideration for a wider range of in vitro and in vivo studies to increase the rigor and reproducibility, and statistical power of the experimental infections that attempt to address some of these key challenges are warranted. After all, the successful development of comparative animal models of human viral infections has important implications in helping to determine the important mechanisms of disease pathogenesis and pathologies and immune correlates of protection against virus infections and/or the diseases that they cause, as well as in informing relevant strategies for vaccination, immunotherapeutic, and antiviral developments to benefit humans.
